# Gastric Antral Vascular Ectasia in Systemic Sclerosis

**DOI:** 10.1155/2011/305238

**Published:** 2011-11-02

**Authors:** Jill Johnson, Chris T. Derk

**Affiliations:** ^1^Rheumatology Division, University of Pennsylvania, One Convention Boulevard, Philadelphia, PA 19104, USA; ^2^Rheumatology Division, Thomas Jefferson University, 1015 Walnut Street, Philadelphia, PA 19107, USA

## Abstract

Gastric antral vascular ectasia is a not so well-understood, and more rare, gastrointestinal manifestation of Systemic Sclerosis which can lead to chronic anemia. A high suspicion and better understanding of this rare manifestation is needed for early detection and treatment. Therapeutic regiments include iron supplementation with acid suppressive therapy, while endoscopic intervention has been shown to be successful in most cases, with gastrectomy or antrectomy rarely needed.

## 1. Introduction

Gastric antral vascular ectasia (GAVE) was first defined in 1984 by Jabbari et al. [1]. Its distinctive endoscopic appearance caused it to be also known as “watermelon stomach.” On visualization it consists of parallel rugal folds with dilated blood vessels going from the gastric antrum and converging on the pylorus resembling watermelon rind and thus the nomenclature. It is often found in association with autoimmune diseases such as diffuse or limited cutaneous systemic sclerosis (dcSSc or lcSSc), Raynaud's phenomenon pernicious anemia, autoimmune hypothyroidism, primary biliary cirrhosis, polymyalgia rheumatic, and rheumatoid arthritis, but it is also found in patients suffering from other chronic medical conditions such as cirrhosis of the liver, chronic renal failure, ischemic or valvular heart disease, hypertension, acute myeloid leukemia, diabetes, and chronic obstructive pulmonary disease [2]. 

## 2. Prevalence and Incidence

GAVE is a rare manifestation of systemic sclerosis. A recent large retrospective study of 264 patients with SSc found a prevalence of 5.7% [3]. Eighty to ninety percent of patients already carry the diagnosis of SSc before GAVE manifests itself [3], but occasionally it can be the initial manifestation of the disease [4, 5]. It is commonly diagnosed early in the course of the disease, often within the first 3 years from diagnosis [3]. Prevalence though may be higher than described because patients who undergo endoscopy are only the ones who are symptomatic or have an unexplained anemia. In a recent abstract from the SCOT trial (Scleroderma cyclophosphamide or transplant study), where asymptomatic dcSSc patients had to be screened with an endoscopy before entering the study, 10.8% of them had silent GAVE [6].

## 3. Risk Factors

In some studies GAVE has been shown to be more prevalent in lcSSc while in other studies in dcSSc patients [3, 7, 8]. Patients with dcSSc tend to develop GAVE earlier in their disease course than patients with lcSSc, often the diagnosis is made within 2 years from the diagnosis of dcSSc, and in a recent series of twenty-eight patients with SSc and GAVE patients with dcSSc were diagnosed at a mean of 21.5 months into their disease course compared to 82.6 months in patients with lcSSc. The patients with lcSSc in this series also tended to have less severe anemia [7]. This may be a reflection of the natural history of lcSSc which has a more indolent course as compared to dcSSc. 

An association between rapidly progressive skin changes and the development of early onset GAVE (i.e., development of GAVE within 18 months of the onset of SSc symptoms) may also exist. A subset of 16 patients from the series of 28 patients in the article by Ingraham et al. were diagnosed with early-onset GAVE. Nine out of these 16 patients also had rapid progression of their cutaneous disease (i.e., the presence of diffuse involvement in the upper extremities and trunk by 18 months from the onset of the first SSc symptoms) [7]. Ceribelli et al. also suggested that rapid progression of cutaneous disease is associated with the early development of GAVE [9]. 

The autoantibody profile may also give clues to the risk of developing GAVE, and a speckled pattern appears to carry a higher risk [7]. A recent report suggests a possible predictive role of RNA polymerase III (RNAP III) antibodies for GAVE. Ceribelli et al. reported 16 SSc patients with positive anti-RNAP III antibodies; 4 out of the 16 (25%) had GAVE [9]. This is considerably higher than the overall prevalence of GAVE in SSc patients [3]. It has been shown in several series that lack of antitopoisomerase I antibodies (anti-scl-70) is associated with an increased risk of GAVE [3, 7, 9]. 

## 4. Pathogenesis

The pathogenesis of GAVE is not fully understood though it has been noted in the past when pathology specimens from GAVE patients were examined that there is a loose attachment of the distal gastric mucosa to the underlying muscularis externa, where in the setting of dysmotility the antral mucosa may prolapse through the pylorus [1, 3, 10]. Pathology specimens show dilated capillaries, focal fibrin thrombi, spindle cell proliferation on the mucosal surface, fibromuscular hyperplasia in the lamina propria, and dilated submucosal vessels [1, 3, 11]. 

GAVE is considered one of the manifestations of the spectrum of vascular alterations in systemic sclerosis [8]. Studies have shown that the majority of patients with GAVE, around 60%, have telangiectasias of the skin. A smaller percentage also has telangiectasias in the gastrointestinal tract other than the stomach, in the esophagus, duodenum, ileum, colon, and rectum [3]. Supporting this theory is the fact that similar histopathologic changes can be found in the dermis on skin biopsies of scleroderma patients as are found in gastric mucosal biopsies from GAVE patients—that is, capillary dilatation, small vessel fibrin deposits, and platelet thrombosis [3, 8]. 

## 5. Clinical Evaluation and Diagnosis

Most patients with lcSSc and GAVE present with iron deficiency anemia or symptoms attributable to iron deficiency anemia, such as weakness, severe fatigue, or dyspnea on exertion. Thus a high index of suspicion is often needed in order to make the diagnosis in a timely fashion. Patients with dcSSc have a tendency to present with occult blood in the stool, and occasionally one may present with overt gastrointestinal bleeding though melena or hematemesis is unusual [3, 10]. Recurrent indolent bleeding is common, occurring in approximately one-third of cases [3]. 

## 6. Endoscopic Description

The appearance of GAVE is longitudinal red stripes or rugal folds in the antrum, radiating out in pattern resembling spokes from the pylorus. Alternatively, multiple cherry-red spots may be seen, but this mosaic pattern is far less common. Antral biopsies typically show capillary dilation with focal intravascular thrombi and fibromuscular hyperplasia in the lamina propria [1]. 

## 7. Management

Management of GAVE ranges from conservative symptomatic therapy to endoscopic interventions to surgery. The mainstays of symptomatic therapy and medical management are iron supplementation, proton pump inhibitors, and blood transfusion if anemia is severe and symptomatic [3]. There are some reports in the literature of treatment with estrogen-progesterone combination hormone therapy, but the risks and benefits must be carefully weighed in each individual case [12–14]. 

Early diagnosis of GAVE in SSc patients is important because endoscopic management can successfully treat this. It is important to educate primary care physicians who take care of these patients to be aware of slight drops in the hemoglobin of SSc patients and to refer such patients for endoscopic evaluation. While lcSSc patients typically have a good outcome after local therapy, dcSSc are more difficult to treat, with a need for recurrent electrocoagulation and blood transfusions and an increased risk for renal crisis.

Several unique endoscopic interventions have been reported to be successful in the management of GAVE. One popular technique is argon plasma coagulation, which has been shown to be effective in short- and medium-term followup in maintaining a rise in hemoglobin and giving transfusion independence to formerly transfusion-dependent patients within 2-3 treatment sessions [15]. Occasionally it can be associated with discomfort from gastric overdistension with argon gas [16]. 

Another endoscopic technique is neodymium-yttrium aluminum garnet (Nd:YAG) laser photocoagulation. A study of 45 patients treated with Nd:YAG laser reported that, at an average of two-year followup, normal hemoglobin levels were seen in 87% of all patients and transfusion independence was found in 24 of 28 initially transfusion-dependent patients [10]. 

There have been case reports describing many other successful therapies such as endoscopic band ligation [17], endoscopic ablation with hot biopsy forceps [16], and endoscopic therapy with monopolar electrocoagulation and injection of 5% polidocanol [18].

 There have been reports of success in two small series of patients using intravenous cyclophosphamide to treat systemic sclerosis-associated GAVE or remission of GAVE when cyclophosphamide is used to treat other serious disease manifestations such as pulmonary disease [19, 20]. 

Gastrectomy or antrectomy is the only cure for GAVE, but it is rarely needed in the modern era with such a vast array of medical and endoscopic therapies available. 

## 8. Conclusion

Gastric antral vascular ectasia continues to be a not well-understood manifestation of SSc patients, but an increase awareness and early diagnosis can improve outcome since local therapy can be successful. There is a need for a noninvasive therapy, though currently the best approach in the care of these patients is close followup and repeated local therapy.
